# First-Principles Simulation of Dielectric Function in Biomolecules

**DOI:** 10.3390/ma14195774

**Published:** 2021-10-02

**Authors:** Puja Adhikari, Rudolf Podgornik, Bahaa Jawad, Wai-Yim Ching

**Affiliations:** 1 Department of Physics and Astronomy, University of Missouri-Kansas City, Kansas City, MO 64110, USA; paz67@umkc.edu (P.A.); bajrmd@mail.umkc.edu (B.J.); 2 School of Physical Sciences, Kavli Institute of Theoretical Science, University of Chinese Academy of Sciences, Beijing 100049, China; 3 CAS Key Laboratory of Soft Matter Physics, Institute of Physics, Chinese Academy of Sciences, Beijing 100090, China; 4 Wenzhou Institute, University of Chinese Academy of Sciences, Wenzhou 325000, China; 5 Department of Physics, Faculty of Mathematics and Physics, University of Ljubljana, SI-1000 Ljubljana, Slovenia

**Keywords:** electrostatic interaction, dielectric function, biomolecules, ab initio simulation, random phase approximation

## Abstract

The dielectric spectra of complex biomolecules reflect the molecular heterogeneity of the proteins and are particularly important for the calculations of electrostatic (Coulomb) and electrodynamic (van der Waals) interactions in protein physics. The dielectric response of the proteins can be decomposed into different components depending on the size, structure, composition, locality, and environment of the protein in general. We present a new robust simulation method anchored in rigorous ab initio quantum mechanical calculations of explicit atomistic models, without any indeterminate parameters to compute and gain insight into the dielectric spectra of small proteins under different conditions. We implement this methodology to a polypeptide RGD-4C (1FUV) in different environments, and the SD1 domain in the spike protein of SARS-COV-2. Two peaks at 5.2–5.7 eV and 14.4–15.2 eV in the dielectric absorption spectra are observed for 1FUV and SD1 in vacuum as well as in their solvated and salted models.

## 1. Introduction

The dielectric properties of proteins in aqueous solutions have been studied for several decades [1] and play a crucial role in the calculations of protein electrostatic Coulomb interactions [2] as well as electrodynamic dispersion van der Waals interactions [3,4], specifically to calculate the protein–protein and protein–nucleic acid interactions, characterize the folding pathways, and investigate solution behavior and stability. In fact, quite recently electrostatic contributions have been invoked as a possible source of the differences between the binding free energy of the spike proteins of SARS-CoV-2 and SARS-CoV to the ACE2 human receptor [5], as well as being the principal interaction component between the spike proteins of SARS-CoV-2 virus and the charged electret fibers in personal protective gear [6].

The problem of the macroscopic protein dielectric “constant” is exacerbated by the fact that, as in other biomolecular systems such as lipid membrane bilayers composed of different segregated molecular components, the underlying microscopic dielectric properties are heterogeneous [7] and the continuum assumptions become—to say the least—problematic on a molecular level [8]. Consequently, the protein dielectric constant appears rather as a phenomenological parameter, or even a phenomenological function of the position inside the protein core [9], that depends on the model used to describe the heterogeneous molecular structure of proteins, then a single universal constant grounded in some fundamental theory [10]. These are important issues as many continuum computational approaches [11] as well as simulation methodologies to characterize protein electrostatics [12] hinge crucially on the numerical choice for the dielectric constant of the protein interior, which seems to matter not only quantitatively but in many cases even qualitatively [13].

Detailed knowledge of the dielectric response in the frequency domain is particularly important for the calculation of the dispersion van der Waals interactions [14], which are expressed as a non-local functional of the dielectric spectrum at imaginary frequencies in the Lifshitz macroscopic theory [3]. Notably, the first computation of van der Waals interaction with full spectral resolution was performed for a biophysical system of interacting phospholipid membranes [15]. Non-covalent dispersion van der Waals interactions are also thought to play an important role in protein folding [16] since they make a substantial contribution to protein–protein as well as protein–water interactions [17]. Additionally, in applications tied to organic optical and electronic devices as well as biotechnology, the contribution of van der Waals interactions to the stability of proteinaceous films deposited on a dielectric substrate has been recently noted [18,19].

Even though the dielectric properties of various metals, semiconductors, and insulators, organic as well as inorganic, have been critically assessed in great detail [14], the dielectric spectrum data for proteins and polypeptides mostly lack the relevant details regarding the optical properties in a wide frequency range from zero frequency to the far ultraviolet [18]. In the absence of relevant experimental data, the calculated ultraviolet frequency region dielectric spectra for the cyclic tripeptide RGD-4C (1FUV) [20], based on an ab initio quantum mechanical (QM) computational scheme, were successfully used to calculate the oscillator strengths, oscillator frequencies, and the relaxation parameters that enter the calculations of the van der Waals interactions in proteinaceous systems [19].

Motivated by the past successful calculations of the electronic part of the dielectric function at full spectral resolution for many crystals and amorphous solids of infinite extent, based on the Bloch Theorem [21] considered to be the cornerstone of the electronic structure theory of condensed matter physics, we now apply the same methodology to proteins where by assumption the unit cell encloses the whole protein. We use the ab initio quantum mechanical (QM)-based computational scheme, dubbed the quantum mechanical random phase approximation (QMRPA) method, anchored in random phase approximation (RPA) [22], to calculate the optical transitions from occupied to unoccupied states with explicit inclusion of the dipole transition matrix from the ab initio wave functions. In what follows, we apply this methodology to calculate the full spectral resolution of the dielectric response for protein molecules, as well as to calculate the relevant partial charges and to generalize the concept of bond order parameter from the usual inter-atomic domain to be able to also characterize the bonding of complete amino acids (AAs). There are of course major differences between the standard implementation of this methodology in solid state physics and its application to biomolecules, specifically to proteins, which are due to their finite size, the fact that they possess no periodically replicated unit cell, and finally that they are usually immersed in a bathing aqueous medium of its own molecular structure. 

Here, we implement the ab initio QMRPA approach to obtain the dielectric spectra, the partial charges and the AA bond order to different biomolecules such as polypeptides and proteins, specifically the small RGD (1FUV) polypeptide in different environments as well as the subdomain SD1 of the SARS-CoV-2 spike protein [23]. Our calculations are free of adjustable parameters and scalable for larger biomolecules. While some ab initio calculations of molecular polarizability in response to an external electric field have been attempted in the past, they were mostly focused restrictively to very small molecules such as single AAs [24,25,26], and may have included electronic as well as vibrational effects. To our knowledge, ab initio calculation has not been attempted for small proteins consisting of a substantial number of different AAs, which are of course far more challenging [27].

## 2. Methods

### 2.1. Model Construction

The starting structure of RGD-4C peptide was downloaded from the RCSB protein data bank (PDB) with ID: 1FUV [28] based on nuclear magnetic resonance (NMR) data. 1FUV contains 11 AAs with 135 atoms, including hydrogen atoms. From the initial dry model, two solvated models with 80 and 100 H_2_O molecules and two salted models with H_2_O and salt ions at concentration 0.15 M were obtained. The solvated models were generated by the three-point charge TIP3P model [29] using LEaP program included in the AMBER 18 package [30].

In total, five RGD-4C models were constructed: one dry, two solvated, and two salted models with monovalent salt at 0.15 M concentration (4 Na^+^ and 3 Cl^−^ ions). These ions are systematically placed in a shell surrounding the protein by using a Coulombic potential on a grid with the program LEaP, while the number of ions is determined from the volume of the cell. For the dry model of SD1 in the spike protein of SARS-CoV-2, its fully relaxed structure was obtained from PDB [23] with ID: 6VSB [31]. The solvated SD1 model is built with 300 water molecules using the same approach as in the 1FUV cases. The selection of 300 water molecules is based on the UCSF Chimera program [32]. All models including 1FUV and SD1 were optimized using VASP.

### 2.2. Structure Optimization Using VASP

This initial atomic-scale structure for the individual small protein is then fully optimized by using the Vienna ab initio simulation package (VASP) [33]. The pseudopotential plane-wave based VASP package is known for its efficiency in relaxation to the equilibrium structure at minimal energy.

Our experience and tests suggest the use of the following input parameters in VASP: Energy cut-off energy at 500 eV, electronic convergence of 10^−4^ eV; force convergence for ionic steps at −10^−2^ eV/Å and a single k-point sampling. After the optimization for SD1 with 24 amino acids and 391 atoms, the total energy decreases from −2370.90 to −2379.21 eV or 2.05 kJ/mol per atom. The VASP-relaxed structure is used for the electronic structure, interatomic bonding and dielectric function calculations using OLCAO method described below.

We used the projector augmented wave (PAW) method with Perdew–Burke–Ernzerhof (PBE) exchange correlation potential [34] within the generalized gradient approximation (GGA).

### 2.3. DFT Calculations Using OLCAO

For electronic structure, interatomic interactions, and dielectric function calculations, a different DFT method is used, the all-electron orthogonalized linear combination of atomic orbitals (OLCAO) method [35], developed in-house. The combination of these two different DFT codes is extremely efficient for large complex materials and is well documented [20,36,37,38] especially for complex proteins such as those of the SARS-CoV-2 virus [23,39,40]. The key feature of the OLCAO method is the possibility it provides to calculate the effective charge ($Q^{*}$) on each atom as well as the bond order values *ρ_αβ_* between any (αβ) pair of atoms.

The partial charge (PC) or ($\Delta Q_{\alpha }=Q_{\alpha }^{0}-Q_{\alpha }^{*}$) is the deviation of the effective charge $Q_{\alpha }^{*}$ from the neutral atomic charge $Q_{\alpha }^{0}$ on the same atom α. The bond order (BO) values are obtained from the ab initio wave functions with atomic basis expansion calculated quantum mechanically:

(1) $$Q_{\alpha }^{*}=\underset{i}{\sum }\underset{m,occ}{\sum }\underset{j,\beta }{\sum }C_{i\alpha }^{*m}C_{j\beta }^{m}S_{i\alpha ,j\beta }\;$$

(2) $$\rho_{\alpha \beta }=\underset{m,occ}{\sum }\underset{i,j}{\sum }C_{i\alpha }^{*m}C_{j\beta }^{m}S_{i\alpha ,j\beta }\;$$

In the above equations, $S_{i\alpha ,j\beta }$ are the overlap integrals between the $i^{th}$ orbital in $\alpha^{th}$ atom and the $j^{th}$ orbital in the $\beta^{th}$ atom. $C_{j\beta }^{m}$ are the eigenvector coefficients of the $m^{th}$ occupied molecular orbital levels. The BO values $\rho_{\alpha \beta }$ in Equation (2) can be calculated for every pair of atoms (α, β) in the optimized structure with precise atomic positions. The BO quantifies the strength of the bond between two atoms and generally scales with the bond length (BL) being also influenced by the surrounding atoms. The sum of BO of all pairs in the system gives the total bond order, TBO. The calculation of PC and BO are based on the Mulliken scheme [41,42], and hence are basis-dependent. Comparisons of BO values using different methods should be treated with caution.

In proteins, the focus is rather on AAs or their individual residues, then on the individual atoms. AAs in fact contain different atoms in different molecular configurations and orientations. Strictly speaking, assigning the distance of separation between two AAs in a protein to describe their interaction is a vague and arbitrary parameter. However, with the quantum mechanically based OLCAO method and with the interatomic interaction between all atoms readily available, we can define the bond order between two AAs *u* and *v* without any ambiguity, and denote it as *amino acid bond pair* (AABP) [40]:

(3) $$\mathrm{AABP}\left(u,v\right)=\underset{\alpha \epsilon u}{\sum }\underset{\beta \epsilon v}{\sum }\rho_{\alpha i,\beta j}\;$$

where the summations are over atoms α in AA u and atoms β in AA v. This is a rigorously defined quantity and can be further extended to different units or (sub)groups of AAs if necessary. The merit of the above scheme is that AABP includes all possible bonding between two amino acids such as covalent, ionic, hydrogen bonding (HB) and even their intermediate mixtures [43].

This single quantitative parameter reflects the internal bonding strength among different AAs in a protein, or AAs between different proteins. While proteins are generally characterized by a primary sequence of nearest neighbor (NN) AAs, non-NN amino acids in the 3D structure of a protein can also interact and the AABP can be resolved into the local NN part and the non-local bonding part, providing much more penetrating details on inter AA bonding.

### 2.4. Optical Transition and Random Phase Approximation

We use OLCAO method to calculate the optical transitions in a biomolecule from occupied to the unoccupied molecular states within the random phase approximation [22]. The transition explicitly involves the dipole transition matrix elements calculated quantum mechanically from the ab initio wave functions which automatically obey the transition rules involved. We name the procedure thus outlined as the quantum mechanical random phase approximation (QMRAP) method.

The complex frequency dependent dielectric function, ε(ω), is given by the standard decomposition:

(4) $$\varepsilon \left(\omega \right)=\varepsilon_{1}\left(\omega \right)+i\varepsilon_{2}\left(\omega \right)\;$$

where

(5) $$\varepsilon_{2}\left(\omega \right)=\frac{e^{2}}{\pi m\omega^{2}}\int_{BZ}dk^{3}\underset{n,l}{\sum }{\left|\langle \Psi_{n}\left(\vec{k},\vec{r}\right)\left|P\right|\Psi_{l}\left(\vec{k},\vec{r}\right)\rangle \right|}^{2}\delta \left(E_{n}\left(\vec{k}\right)-E_{l}\left(\vec{k}\right)-E\right)$$

Here, $\psi_{n}\left(\vec{k},\vec{r}\right)$ is the Bloch wave function for the $n^{th}$ band with energy $E_{n}\left(\vec{k}\right)$ at Brillouin zone point k. Momentum matrix elements $\langle \Psi_{n}\left(\vec{k},\vec{r}\right)\left|P\right|\Psi_{l}\left(\vec{k},\vec{r}\right)\rangle$ from occupied valence band states (l) to empty conduction band states (n) are calculated from ab initio wave functions. The $E_{l}$ and $E_{n}$ are the energy of occupied state and unoccupied state, respectively. E=ℏω is the photon energy.

The real part $\varepsilon_{1}\left(\omega \right)$ can be derived from $\varepsilon_{2}$ by using the Kramers–Kronig relation [44]:

(6) $$\varepsilon_{1}\left(\omega \right)=1+\frac{2}{\pi }P\int_{0}^{\infty }\frac{s\varepsilon_{2}\left(s\right)}{s^{2}-\omega^{2}}ds,$$

while the energy loss function F(ω) can be obtained as:

(7) $$F\left(\omega \right)=IM\;\left(-\frac{1}{\varepsilon \left(\omega \right)}\right)=\frac{\varepsilon_{2}\left(\omega \right)}{\varepsilon_{1}^{2}\left(\omega \right)+\varepsilon_{2}^{2}\left(\omega \right)}$$

In crystalline solids, the position of the peak of the energy loss function F(ω) at $\omega_{0}$ is identified as the plasmon frequency, which is the energy for the collective excitation of the electrons in the unoccupied bands.

### 2.5. Past Record of Using above Methods

The QMRPA method for inorganic crystalline and amorphous materials has been successfully used by us over the last 30 years [35]. Notable examples are the timely calculations of the dielectric functions of two exceptional materials, the high temperature ceramic superconductor YBa_2_Cu_3_O_7−δ_ (YBCO) in 1987 [45,46] and the Buckminster fullerene C_60_ FCC crystals in 1991 [47,48]. More recent examples of complex materials include mixed inorganic glass (a-SiO_2_)_1−x_(GeO_2_)_x_ [49] and amorphous zeolitic imidazolate framework (a-ZIF) showing the metal–insulator transition under compression [50,51].

The calculation of dielectric functions of biomaterials started nearly 20 years ago. For example, the calculation of optical transition in vitamin B_12_ Adenosyl-cobalamin has shown good agreement with experimental spectra [52,53]. Similar calculations of the electronic structure and X-ray absorption spectra were carried out on hydroxyapatite and different calcium apatite crystals [54,55]. A particularly interesting example is the calculation of the imaginary part of the dielectric function ε_2_(*ω*) of herapathite, a large complex dichroic crystal (C_20_H_24_N_2_O_2_H_2_)_4_·C_2_H_4_O_2_·3SO_4_·2I_3_·6H_2_O [56], verifying the strong linear dichroism with anisotropy factor of 385 that has numerous applications for its polarizing properties [57].

Other examples include the application of QMRPA method to dispersion interactions based on dielectric spectra for biomolecular systems [58,59,60] and the effect of optical absorption of cytochrome c (Cyt c) macromolecules at the interface or surface for high performance photodetection [61,62]. Last but not the least is the special peptide (RGD-4C) that will be highlighted in later sections. In the present paper, we focus on the QMRPA method as applied to the RGD (1FUV) polypeptide.

## 3. Results on RGD (1FUV) Peptide

This section focuses on the calculation of electronic structure, dielectric function, and partial charge of a simpler biomolecule RGD (1FUV) peptide as a specific example. Other results including the extension to a more complex biomolecule SD1 domain of spike protein in SARS-CoV-2 and other relevant properties are separately described in Section 4 and Section 5 that follow.

### 3.1. RGD (1FUV) Peptide

Peptide sequences with the arginine-glycine-aspartic acid (RGD) motif display a strong affinity and selectivity for a membrane protein called *integrin*, which is a key cell surface receptor mediating cell adhesion to extracellular matrices (ECM) [63,64]. Integrins are actively expressed on vascular endothelial cell surfaces and play a key role in tumor metastasis, leukocyte migration, and angiogenesis [65], making them an ideal target for treating inflammatory diseases and cancer. Because integrin receptors recognize RGD as a primary sequence, RGD peptides are used to target cancer cells [66]. In addition, RGD peptides provide numerous applications in biological and biomedical devices, being frequently incorporated into biomaterials designed to facilitate wound healing [67], serving as candidates for radiotracers in imaging [68], being used in implantable medical devices [69], and mimicking the activities of the ECM proteins in culture cells [70].

We use the QMRPA method to calculate the dielectric function of a cyclic tripeptide RGD-4C to demonstrate its applicability in the case of a small protein. The initial structure of the RGD-4C was obtained from the Protein data bank (PDB ID: 1FUV) based on nuclear magnetic resonance (NMR) data [28]. The electronic structure, dielectric response, and the solvent accessible surface partial charge distribution of the RGD peptide has been studied already [20], while here we present improved accuracy calculations of the dielectric function of dry 1FUV as well as for the 1FUV solvated and salted models.

**Figure 1:**
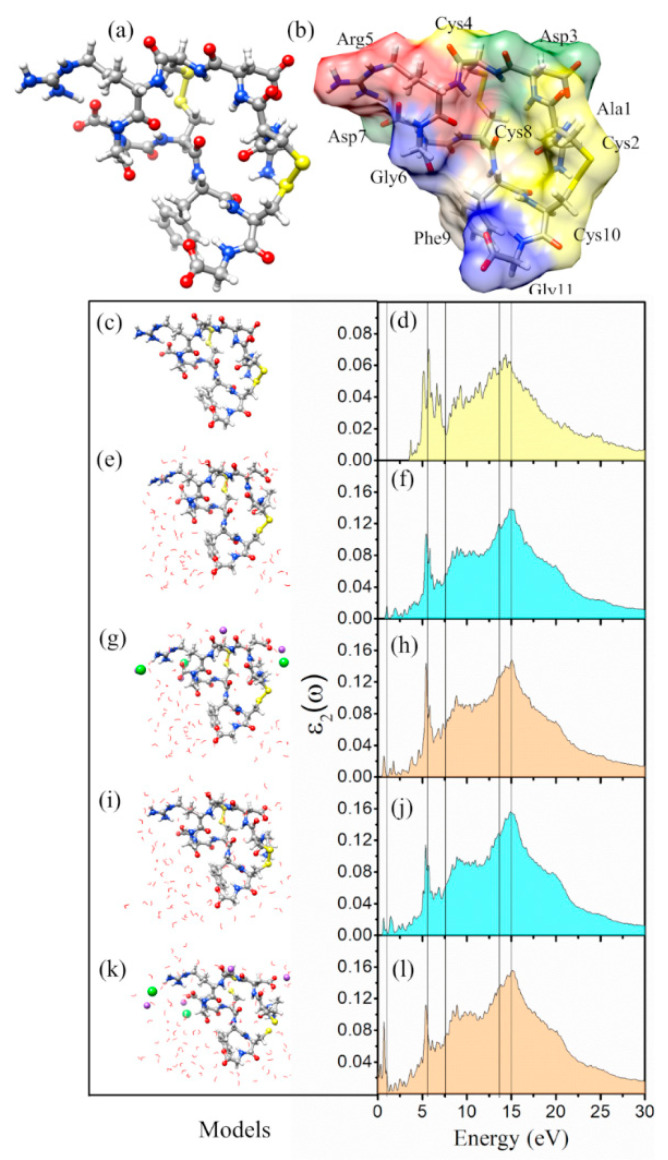
Dry, solvated, and salted models for 1FUV with their $\varepsilon_{2}\left(\omega \right)$ spectra and $\varepsilon_{1}\left(0\right)$ values. Ball and stick illustration of (**a**,**c**) dry 1FUV with (**b**) surface shown with different colors. $\varepsilon_{2}\left(\omega \right)$ for: (**d**) dry 1FUV; (**f**) 1FUV with 80 H_2_O, (**h**) 1FUV with 80 H_2_O and 0.15 NaCl, (**j**). 1FUV with 100 H_2_O, and (**l**) 1FUV with 100 H_2_O and 0.15 NaCl. The ball and stick figure for respective cases are in (**e**,**g**,**i**,**k**) the left panel. Gray: C, red: O, blue: N, white: H, green: Cl, and purple: Na.

1FUV has 11 AAs (ACDCRGDCFCG) of six different types: Gly (two), Cys (four), Phe (one), Asp (two), Arg (one), and Ala (one). For its 135 atoms, including H atoms, it is somewhat clustered but otherwise a rather typical small peptide. It is one of the most used RGD variants because of the presence of the four Cys residues which allow for the two rare disulfide bonds. Figure 1a–c show a ball and stick structure of 1FUV after VASP relaxation. The 11 AAs in 1FUV are marked in Figure 1b. There are considerable non-local interactions between non-nearest neighbor (NN) AAs in the primary sequence. This aspect of 1FUV will be elaborated further in Section 5.

Figure 1d shows the final imaginary part $\varepsilon_{2}\left(\omega \right)$ for up to 30 eV in the case of dry 1FUV and will be discussed again in the following section together with the spectra (Figure 1e,f,i,j) for the solvated and (Figure 1g,h,k,l) for solvated and salted models. As can be seen, the general feature of $\varepsilon_{2}\left(\omega \right)$ of 1FUV is the broader peak at 14.4 eV and discernable multiple sharp peaks below 5.7 eV. We now proceed to the solvated and salted models with 80 and 100 water molecules and added 0.15 M NaCl (4 Na and 3 Cl ions).

### 3.2. Local Environmental Effect on RGD (1FUV) Peptide

For biomolecular systems and proteins in particular, environment effects such as the presence of solvent, fixed pH reservoir, fixed salt content and *protonation/deprotonation equilibrium* etc. must ideally be considered. In the explicit QMRPA calculation, which is a single point calculation, a specific model for each of the environmental effects needs to be constructed individually with increased size and complexity.

The construction of four solvated and salted models is described in the Method Section 2.1. The atomic structures of these four solvated models are further optimized using VASP and the final ball and stick sketch for these four models are displayed in the left panel of Figure 1. These 2D projections of the solvated structures of 1FUV clearly show that the water molecules are evenly distributed with random orientation and no penetration of water into the interior of the protein. The locations of the Na^+^ and Cl^−^ ions depend on the VASP optimization routines, corresponding to the most energetically favorable positions.

**Figure 2:**
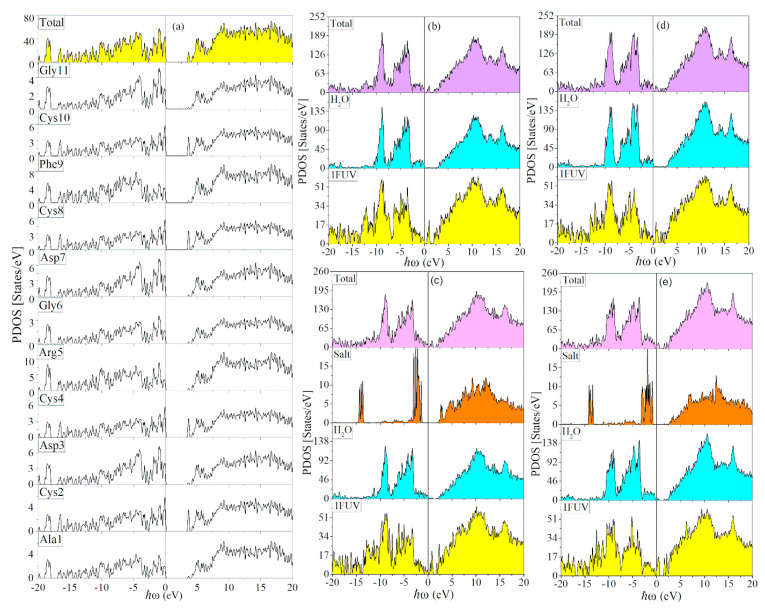
DOS and PDOS of 5 models for 1FUV: (**a**) Dry model further resolved for each amino acid in a separate panel; (**b**) with 80 water molecules resolved into 1FUV and 80 H_2_O; (**c**) with 80 H_2_O molecules, and 4 Na and 3 Cl ions resolved into 1FUV, 80 H_2_O, and 0.15 NaCl (**d**) with 100 water molecules resolved similar to (**b**,**e**) with 100 H_2_O molecules, and 0.15 NaCl resolved similar to (**c**).

We now present the results for these four solvated and salted 1FUV models together with the dry model. The calculated dielectric spectra $\varepsilon_{2}\left(\omega \right)$ of the four solvated 1FUV models are shown in Figure 1f,h,j,l. It can be seen that the general features in the solvated models are similar to the dry model in Figure 1d, except for the appearance of many small transition peaks below 6 eV arising from the transitions from or to the gap states, as evidenced in the density of states (DOS) presented in Figure 2. When the salts Na^+^ and Cl^−^ ions are added, the $\varepsilon_{2}\left(\omega \right)$ and $\varepsilon_{1}\left(\omega \right)$ spectra are changed even further in a more complex manner depending on the actual location of the gap states and the strength of their dipole transition matrix elements (Equation (5) in Methods Section 2.4).

Figure 2 shows the calculated DOS and partial DOS (PDOS) for all five models. For the dry 1FUV, the total DOS (TDOS) is also resolved into 11 different AAs. The four S-containing Cys residues have separate peaks in the LUMO, which is not the case for other individual AAs. This feature comes from the interaction of Cys with the other AAs in 1FUV. We also observe that there are no *gap states* between the HOMO-LUMO gap in the dried 1FUV model as shown in Figure 2a. In addition, dry 1FUV has a wider band gap (Eg) in comparison to solvated and salted models as shown in Table 1. In Figure 2b–e for the solvated and salted models, the PDOS are resolved into those from 1FUV, the H_2_O molecules and the salt ions. It is of interest to note that the TDOS in Figure 2b–e does have many gap states, but the PDOS can show it arises due to the interaction between different AAs in solvated 1FUV, the individual H_2_Os and the Na^+^ or Cl^−^ ions of the dissolved salts.

**Figure 3:**
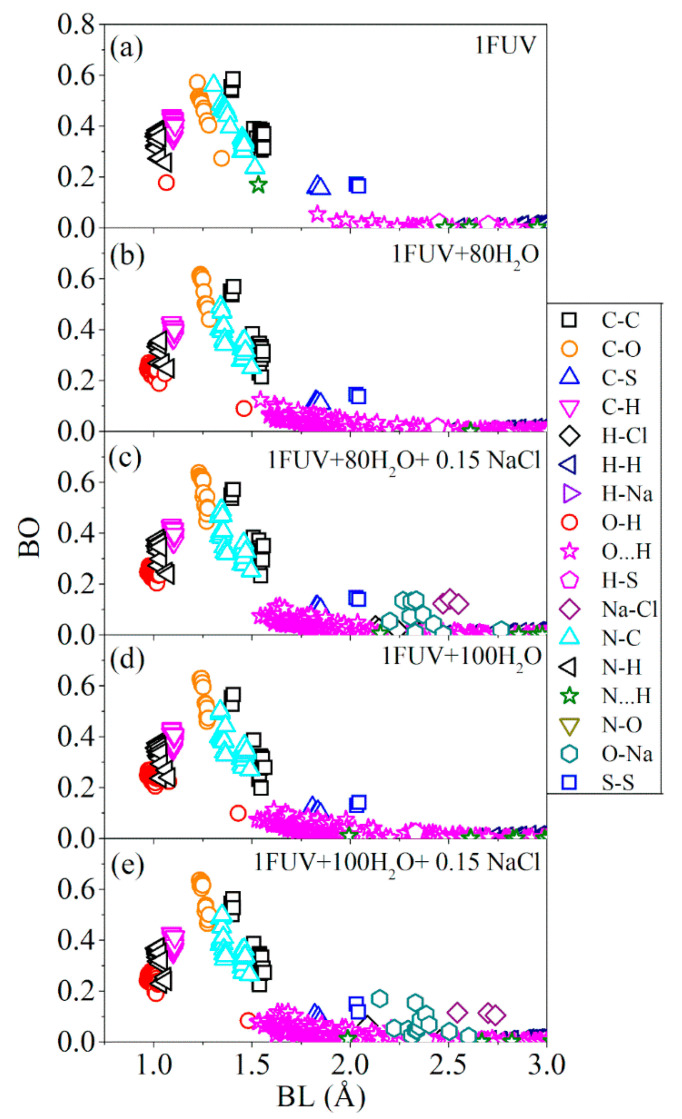
Distribution of BO vs. BL in the three 1FUV models: (**a**) dry 1FUV; (**b**) 1FUV with 80 H_2_O, (**c**) 1FUV with 80 H_2_O and 0.15 NaCl; (**d**). 1FUV with 100 H_2_O, and (**e**) 1FUV with 100 H_2_O and 0.15 NaCl.

The bond order (BO) for every pair of atoms calculated from Equation (2) in Method Section 2.3), is an integral part of the description of biomolecular materials [35,39,40]. Figure 3 shows BO versus bond length (BL) plots for the dry 1FUV model and the more complex solvated model with 80 and 100 H_2_O molecules and salted models with 80 H_2_O and 100 H_2_O molecules. The bond order includes contributions within each AA, between different AAs, and between atoms in each of the AA with H_2_O molecules or salt ions in the solvated models.

For clarity, we briefly describe the dry 1FUV model and the solvated and salted 1FUV model with 100 H_2_O molecules and salt atoms of Figure 3.

*Dry 1FUV:* the strongest bonds are of course the covalent bonds containing C (C-C, C-O, N-C, C-H). Their BL varies slightly but BO values can vary rapidly, scaling with BL. The BO depends on the nature of the covalent bond (single or double), the atoms it bonds and, to a lesser extent, the local environment of the bonded pair. The covalent and C-H bonds within or between different AAs have a BL slightly larger than 1.0 Å and exhibit varying BO values. In Figure 3a, there is a singular O-H bond that can be traced to O in residue Asp7 and H in residue Arg5 which can also be interpreted as an exceptionally strong HB. The hydrogen bonds (HB) O∙∙∙H or N∙∙∙H are of vital importance in any biological system, being weaker than covalent bonds but ubiquitous especially in the presence of solvents. The BLs of HBs generally range between 1.5 and 3.0 Å and can have BO values larger than 0.1 e^−^ in some cases. Figure 3a shows the presence of a very strong N∙∙∙H bond with a BO of 0.016 e^−^ and a BL of 1.532 Å with H and N, pertaining to the same residue Arg5. The rest of the HBs are all O∙∙∙H bonds with a much longer BL above 1.6 Å. The other important bonds are the two S-S bonds near 2.2 Å and short C-S covalent bonds at 1.75 Å involving S atom in Cys residues. These are all very stable bonds with BO values of 0.16 e^−^ in different environments.

*Solvated 1FUV with 100 H_2_O and salt ions:*Figure 3e shows the BO vs. BL plots for the largest and more complicated 1FUV model with 100 H_2_O molecules and 7 salt ions (4 Na and 3 Cl). The main difference is the appearance of the internal O-H covalent bond in H_2_O together with the new bonds (H-Cl, H-Na, Na-Cl and O-Cl) with the salt ions at much larger BL. Other changes are less remarkable, but it is notable that the single O-H bond and the strong N∙∙∙H HB in Figure 3e have both disappeared, showing the changes in the atomic positions in residues Asp and Arg due to solvation. The appearance of many more O∙∙∙H HBs with shorter HB distances is expected. In addition, there are some minor changes in the N-C bonds while the C-S and S-S bonds become weaker but remain at similar separations. The C-H bonds retain similar separation since the AAs in 1FUV remain relatively intact. The number of O-H bonds is observed to increase with concomitant increase in the BO values. This could imply the possible occurrence of protonation when some H atoms in water molecules rearrange themselves under increased complexity of the environment, although we do not yet have solid evidence for this interpretation. All these larger number of interactions mentioned above lead to its higher total bond order (TBO) in comparison to other four 1FUV models (shown in Table 1).

The ab initio quantum mechanical calculation allows for a very detailed analysis of the interatomic bonding in dry as well as solvated 1FUV, directly affecting the electronic portion of the dielectric response of proteins. Quantitative evaluation of the HB in solvated model is extremely important and universally agreed by all researchers in biomolecular science, yet few have gone deep enough in the search for quantitative details, mostly characterizing the HBs qualitatively based on the separations between H and O or N without quantitative value for their bond strength, especially those using classical molecular dynamic methodology.

### 3.3. Role of Partial Charge

In addition to the dielectric spectra, the partial charge (PC) distribution is also crucial for determining and elucidating the electrostatic Coulomb interactions that play a major role in catalysis [71], drug engineering [72], etc. For different models studied here, the PC values are calculated based on the reliable orthogonalized linear combination of atomic orbitals (OLCAO) methodology [35] to understand the electrostatic effects but also the impact due to aqueous environments. We discuss here the comparison of PC distributions in the five 1FUV models under different local environment.

**Figure 4:**
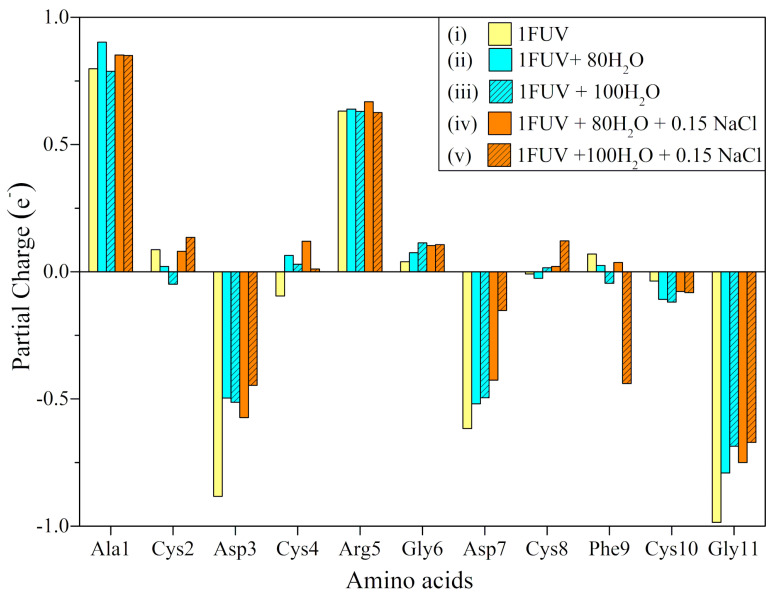
Partial charge (PC) distributions in terms of AAs for five 1FUV models (**i**)–(**v**) in various environments as shown in legends.

The calculated PCs on each of the 11 AAs in the five 1FUV models ((i) dry, (ii) solvated with 80 H_2_O (iii) 100 H_2_O, (iv) salted model of (ii), and (v) salted model of (iii) are displayed in Figure 4. It can be observed that the variations of PC in five 1FUV models are minor for all amino acids with the exception of the negatively charged AAs (Asp3, Asp7) and Gly11, largely affected by the presence of the aqueous solution (water and ions). In particular, Phe9 residue in salted models with 100 H_2_O or pure 100 H_2_O (models (v) and (iii)) differs from other models in which its PC gains a small charge. This behavior is even more pronounced in model (v) when the PC of Phe9 is flipped from a negligible 0.07 e^−^ in the dry case to a negative value of −0.44 e^−^. A close examination of such behavior reveals that the oxygen atom in the backbone of Phe9 can form a HB with one water molecule in all four solvent models, but its separation distance varies. In model (v), this HB has a much shorter distance of 1.65 Å when compared to 1.83, 1.78, and 2.52 Å for models (ii), (iii), and (iv), respectively.

This finding clearly indicates that local environments have a direct effect on PC distributions. It is noteworthy that positive and negative PCs in Ala1 and the Gly11 at both terminals of 1FUV result from the NH_2_^+^ in N-terminal and COO^−^ in C-terminal, respectively, and these opposite PCs neutralize each other.

## 4. SD1 of SARS-CoV-2 Spike-Protein

The last example for the calculation of the dielectric function in proteins is a part of the spike protein in SARS-CoV-2 virus. The spike protein of SARS-CoV-2 is an ideal target for vaccine development and other therapeutic treatment due to its essential role in the virus life cycle [73,74]. The structure of spike protein is very complex as it exists in a trimeric form with each protomer consisting of two functional subunits, S1 and S2. The S1 subunit comprises a signal sequence (SS) at the N-terminal end, followed by NTD and RBD and two structurally conserved subdomains (SD1 and SD2). The S2 subunit is subdivided into a fusion peptide (FP), two heptad repeats (HR1 and 2), and a central helix (CH), a connector domain (CD), a transmembrane domain (TM), and the cytoplasmic tail (CT). Between the S1 and S2 subunits, there are two protease cleavage sites (S1/S2 and S2′) [31].

**Figure 5:**
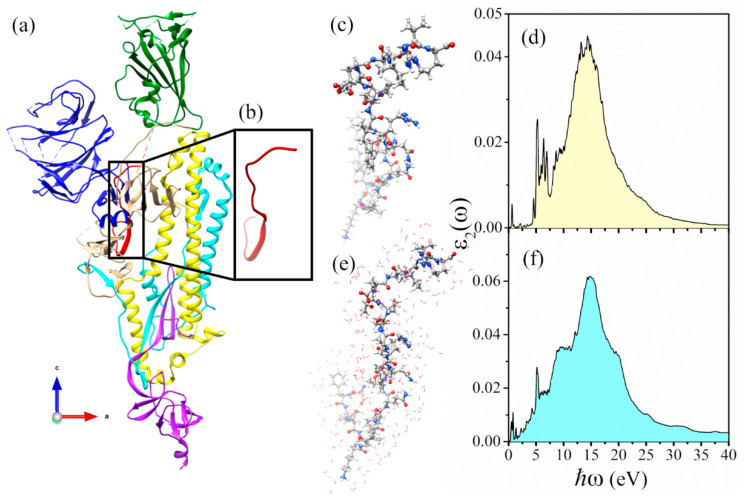
(**a**) Chain A of spike protein (6VSB); (**b**) ribbon for SD1; (**c**) ball and stick model of SD1; (**d**) ε_2_(*ω*) for dry SD1 model; (**e**) ball and stick model and (**f**) ε_2_(*ω*) of SD1 with 300 H_2_O molecules.

The ab initio calculation of the electronic structure and interatomic bonding of this large biomolecular system has been already discussed [23]. SD1 is the smallest of the seven structural domains, having 24 AAs and 391 atoms in the dry environment. Unlike 1FUV, it has an elongated structure mimicking a very irregularly shaped mini-protein as shown in Figure 5a–c. It is therefore highly instructive to apply the QMRPA method to investigate its dielectric spectra of SD1. Other information on the electronic structure and intra- and inter-atomic bonding of SD1can be found in Ref. [23].

The solvation effects in SD1 have been addressed by adding 300 vicinal water molecules, as illustrated in Figure 5e. The solvated structure is then fully optimized by using VASP in the same way as the solvated model of 1FUV. The calculated dielectric absorption spectra for the dry and solvated SD1 are shown in Figure 5d,f. By comparing the dielectric spectra in the case of dry and solvated 1FUV models, shown in Figure 1d,j, we note the occurrence of a broad peak at 14.4–15.2 eV originating from covalent interatomic bonding. In addition, we observe a peak at 5.2–5.7 eV, in cases of dry and solvated 1FUV and SD1 models.

## 5. Amino Acid Bond Pairing—AABP

All polypeptides are composed of an AA primary sequence. Because of the unique 3D structure of folded proteins, the AAs interact not only between nearest neighbors (NN) in the primary sequence, but also with other non-NN or off-diagonal or spatially not vicinal, i.e., non-local AAs. These non-local interactions between AAs along the primary sequence allow for the formation of AA–AA bond pairs (AABPs) (see Equation (3) in Method Section 2.3) [23,40].

**Figure 6:**
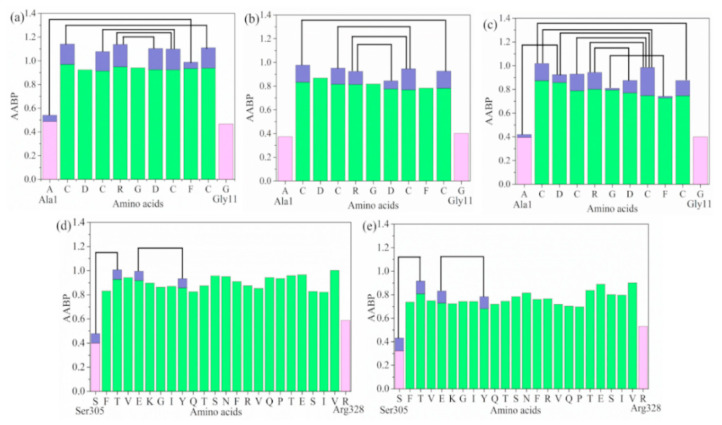
Distribution of AABP in (**a**) 1FUV; (**b**) 1FUV in 80 H_2_O molecules; (**c**) 1FUV in 100 H_2_O molecules; (**d**) SD1, and (**e**) SD1 in 300 H_2_O molecules. The color bar represents the following cases, light pink: sum AABP of AA with single nearest neighbor, green: sum AABP of AA with two nearest neighbors, light blue: AA with off-diagonal AABP. The black curve lines represent off-diagonal bonding between two AAs.

The distribution of AABP and their characteristics in the seven domains of the spike protein in SARS-CoV-2 has been described in detail [23]. The same type of analysis is also performed for the 1FUV protein models and for the SD1 small protein models. Figure 6 shows the comparison of the calculated AABP distribution and the 3D bonding network in dry and solvated 1FUV and SD1. They differ substantially, mainly because of the differences in their structures. SD1 has a long, ribbon-like structure and naturally exhibits less off-diagonal AABP contribution (4 out of 24 AAs or 0.16%), while 1FUV is a rather compact protein with only 11 AAs, displaying a substantial contribution from AABP (8 out of 11 or 73%).

In Figure 6, we display and compare the results for the AABP values in dry and solvated modes for 1FUV and SD1. The main observations can be summarized as follows:

1. The AAs in 1FUV models have far more off-diagonal contributions to AABP than the AAs in SD1, as expected from the very different 3D structure of these two proteins.
2. The solvated models exhibit a reduced total AABP, mainly from the reduced NN interactions.
3. The five off-diagonal pairs in dry 1FUV are reduced to four pairs when solvated by 80 H_2_O molecules. The missing pair is between Ala1-Phe9. Surprisingly, when solvated by 100 H_2_O molecules, the number of pairs increases again to eight. The new pairs are Ala1-Asp3, Cys2-Cys8, Asp3-Cys8, Gly6-Phe9.
4. For SD1, the two off-diagonal pairs remain the same when solvated by 300 H_2_O molecules, but the total AABP values for all AAs are decreased by about 13% on average.
5. The decrease in total AABP in solvated models in comparison to the dry model is due to the interactions between AAs modified by the presence of H_2_O molecules.
6. It can then be concluded that the total AABP values can be changed in a rather complicated fashion depending on the nature of the protein and the amount of water molecules surrounding them.

## 6. Discussion

There is a growing interest in determining the details of the dielectric properties of proteins to better understand and estimate the molecular interactions involving proteins and polypeptides. However, the details of the dielectric response function depend on various factors such as the nature, size, and structure of the protein. In the present study, we report on the implementation of the QMRPA scheme to calculate the dielectric spectra of various biomolecular systems such as 1FUV in five different environments and SD1 of SARS-CoV-2 spike protein in dry and solvated cases.

The present approach using ab initio calculations for the dielectric spectra in proteins can provide input spectral information for the calculation of the dispersion van der Waals interactions, while the bond order and the partial charge calculations can yield parametrizations for the electrostatic Coulomb interactions. Problems related to protein folding and stability, conformational change, mutation, glycosylation, etc., could be elucidated on a more solid basis involving molecular and atomic interactions that can be quantified by the ab initio calculations. However, the application of QMRPA methodology to larger proteins, while challenging due to computational limitations, is certainly not impossible. It is much more effective to use a QMRPA method to a selected number of specific larger macromolecules or semi-macromolecules to provide more accurate parameter-free data than just relying on standard atomic potential parametrizations.

Recently, we have succeeded in the largest ab initio quantum chemical computation to date on the S-protein by using a *divide and conquer* strategy for all subdomains of the spike protein available in 6VSB [23], while here the electronic dielectric properties of SD1, that act as a hinge point for the RBD in the down to up transitions [75] in dry and solvated environments have been investigated. The same type of calculations for other much larger subdomains are also possible to gain additional insights on the electrostatic interactions in the spike protein, and the results could be very useful for COVID-19 researchers working on detection techniques.

For example, a biosensor based on imaging ellipsometry was used to detect two neutralizing monoclonal antibodies and serial serum samples from SARS-CoV patients [76,77]. Ellipsometry is an optical technique closely related to the dielectric properties [77]. We speculate that the calculated dielectric spectra of the spike protein can be helpful in their design and implementation. On other hand, the electrostatic interactions have been demonstrated to play a major role in enhancing the binding affinity of RBD of SARS-CoV-2 to ACE2 as compared to SARS-CoV [78,79,80,81] and the polar solvation free energy of RBD-ACE2 complex is in fact quite sensitive to the dielectric spectra.

Our work on the RBD-ACE2 interface complex is in progress and will be reported elsewhere. We have high expectations that the calculated dielectric spectra based on a more realistic ab initio approach could be used to achieve a reasonable value for overall electrostatic interactions (Coulombic and polar solvation energies) as compared to the experimental data.

## 7. Conclusions

In conclusion, we have presented the dielectric spectra for two small proteins, 1FUV and SD1, where our results indicate that the dielectric absorption spectra show distinct peaks at around 5 and 15 eV. The peaks at 15 eV are a more generic peak from the covalent interatomic bonding, whereas peaks at around 5 eV are more unique but are observed in both biomolecules. A succinct summary of the work presented above is as follows:

1. We introduced the QMRPA method for dielectric spectra for small proteins such as RGD (1FUV) peptide and the SD1 subdomain of the spike protein of SARS-CoV-2 virus.
2. We pointed out the possible connections between atomic scale partial charges of AAs in proteins and their specific role in the electrostatic interaction.
3. We described the role of non-local AA-AA interactions via AABP values in the 3D structure of the protein comparing dry and solvated models.
4. We laid out the roadmap to use QMRPA method for applications to electrostatic interactions in spike protein and other biomolecular systems in general.

## Figures and Tables

**Table 1:** Total bond order (TBO) and band gap (Eg) for five 1FUV models.

Models	TBO (e)	Eg(eV)
1FUV	54.27	3.61
1FUV + 80 H_2_O	98.49	0.68
1FUV + 80 H_2_O + NaCl	99.95	0.47
1FUV + 100 H_2_O	109.64	0.57
1FUV + 100 H_2_O + NaCl	110.30	0.15

## Data Availability

Not applicable.
